# Osteocrin, a novel myokine, prevents diabetic cardiomyopathy via restoring proteasomal activity

**DOI:** 10.1038/s41419-021-03922-2

**Published:** 2021-06-16

**Authors:** Xin Zhang, Can Hu, Xiao-Pin Yuan, Yu-Pei Yuan, Peng Song, Chun-Yan Kong, Teng Teng, Min Hu, Si-Chi Xu, Zhen-Guo Ma, Qi-Zhu Tang

**Affiliations:** 1 Department of Cardiology, Renmin Hospital of Wuhan University, 430060 Wuhan, China; 2 Hubei Key Laboratory of Metabolic and Chronic Diseases, 430060 Wuhan, China

**Keywords:** Proteasome, Heart failure

## Abstract

Proteasomal activity is compromised in diabetic hearts that contributes to proteotoxic stresses and cardiac dysfunction. Osteocrin (OSTN) acts as a novel exercise-responsive myokine and is implicated in various cardiac diseases. Herein, we aim to investigate the role and underlying molecular basis of OSTN in diabetic cardiomyopathy (DCM). Mice received a single intravenous injection of the cardiotrophic adeno-associated virus serotype 9 to overexpress OSTN in the heart and then were exposed to intraperitoneal injections of streptozotocin (STZ, 50 mg/kg) for consecutive 5 days to generate diabetic models. Neonatal rat cardiomyocytes were isolated and stimulated with high glucose to verify the role of OSTN in vitro. OSTN expression was reduced by protein kinase B/forkhead box O1 dephosphorylation in diabetic hearts, while its overexpression significantly attenuated cardiac injury and dysfunction in mice with STZ treatment. Besides, OSTN incubation prevented, whereas OSTN silence aggravated cardiomyocyte apoptosis and injury upon hyperglycemic stimulation in vitro. Mechanistically, OSTN treatment restored protein kinase G (PKG)-dependent proteasomal function, and PKG or proteasome inhibition abrogated the protective effects of OSTN in vivo and in vitro. Furthermore, OSTN replenishment was sufficient to prevent the progression of pre-established DCM and had synergistic cardioprotection with sildenafil. OSTN protects against DCM via restoring PKG-dependent proteasomal activity and it is a promising therapeutic target to treat DCM.

## Introduction

Diabetic cardiomyopathy (DCM) is defined as cardiac structural and functional abnormalities among diabetic individuals independent of hypertension or coronary artery disease, which is mainly characterized as massive cardiomyocyte dropout, interstitial fibrosis, and ventricular dysfunction [1, 2]. It is reported as the key pathogenic factor for heart failure and contributes to more than half of diabetic death [3]. Unfortunately, diabetes-related cardiac dysfunction cannot be effectively improved after strict glycemic control, while the risks of cardiovascular events and heart failure hospitalizations are even increased in diabetic patients with certain glucose-lowering drugs (e.g., rosiglitazone) treatment [4–6]. Therefore, clarifying the pathogenesis of DCM and developing novel cardioprotective strategies are greatly needed.

Proteostasis is fundamental for cellular function and organismal health, and the proteasome plays pivotal roles in protein quality control via removing the damaged proteins timely and accurately [7]. However, cardiac proteasomal activity is reported to be compromised upon different stimulations (e.g., pressure overload, ischemia–reperfusion, and doxorubicin), accompanied by an excessive accumulation of misfolded, oxidized, or other damaged proteins. These proteins subsequently provoke cell injury and apoptosis and accelerate cardiac dysfunction [8–11]. Adult cardiomyocytes are particularly vulnerable to proteotoxic stresses due to the negligible regenerative capability [12]. For this reason, proteasome inhibitors' application during tumor chemotherapy often correlates with a certain risk of cardiotoxicity and heart failure [13, 14]. Besides, Li et al. previously observed that the proteasomal function was progressively impaired in murine hearts soon after the onset of diabetes, while enhancing proteasomal activity was sufficient to diminish diabetes-induced proteotoxic injury and cardiac dysfunction [15]. These compelling evidences define the proteasome as a promising target to treat DCM.

Regular physical activity stimulates multiple cardioprotective adaptions to diabetic individuals and is recommended as the cornerstone of nonpharmacological management for DCM [16–19]. Nevertheless, the exercise capacity is markedly compromised in diabetic patients due to muscle atrophy, toe deformity, motor neuropathy, and cardiopulmonary dysfunction [20–22]. Besides, inappropriate exercise also causes additional traumas and increases the risks of adverse cardio-cerebrovascular events or sudden death in certain diabetic populations [23]. Myokines are kinds of exercise-responsive peptides or cytokines from muscle fibers and mediate diverse cardiac benefits of physical activity [24–29]. Previous findings implied that the myokine, fibroblast growth factor 21 obviously prevented cardiac lipotoxicity and dysfunction in diabetic mice [30, 31]. A novel myokine, irisin suppressed endothelial-to-mesenchymal transition and cardiac fibrosis upon hyperglycemic stimulation [32]. Besides, Liu et al. found that cardiac-specific overexpression of a myokine, mitsugumin 53 caused severe lipotoxicity and maladaptive structural remodeling, thereby driving DCM progression [33]. Thus, targeting these myokines may provide novel cardioprotective methods for diabetic patients, especially for whom cannot tolerate exercise. Osteocrin (OSTN, also known as musclin) is identified as an activity-stimulated myokine and acts to regulate neuronal function, bone growth, and physical endurance [34, 35]. Chiba et al. observed that OSTN was abundantly expressed in the myocardium and could be secreted from the cardiomyocytes [36]. And OSTN overexpression notably prevented inflammatory response, cardiac rupture, and heart failure in infarcted mice [37]. Moreover, our recent findings also determined the therapeutic potential of OSTN on doxorubicin-induced cardiotoxicity [26]. Herein, we sought to investigate whether OSTN could ameliorate DCM and elucidate the potential mechanisms.

## Materials and methods

### Reagents and antibodies

Streptozotocin (STZ) and sildenafil citrate were purchased from Sigma-Aldrich (St. Louis, USA). Carrier-free recombinant human OSTN protein (rhOSTN) was obtained from R&D Systems (Minneapolis, USA). Bortezomib (BZM) and carfilzomib (CFZ), two clinical proteasome inhibitors, were purchased from Selleck Chemicals (Houston, Texas, USA). A selective inhibitor for protein kinase G (PKG), KT5823 and the lactate dehydrogenase (LDH) assay kit were obtained from Abcam (Cambridge, UK). EnzChek™ caspase3 assay kit was purchased from ThermoFisher Scientific (Waltham, USA). Proteasomal activity detecting kit, Suc-Leu-Leu-Val-Tyr-AMC, Z-Leu-Leu-Glu-AMC, and Boc-Leu-Arg-Arg-AMC were obtained from UBPBio (Aurora, USA). Cyclic GMP (cGMP) XP^®^ assay kit was obtained from Cell Signaling Technology (Danvers, USA), while cGMP-dependent protein kinase assay kit was purchased from CycLex^®^ (Nagano, Japan). Terminal deoxynucleotidyl transferase-mediated dUTP-biotin nick end labeling (TUNEL) assay kit was purchased from Millipore (Billerica, USA) and the cell counting kit-8 (CCK-8) was obtained from Dōjindo Laboratories (Kumamoto, Japan). Anti-B-cell lymphoma 2 (BCL-2) was obtained from Abcam (Cambridge, UK), whereas primary antibodies against the following proteins were purchased from Cell Signaling Technology (Danvers, USA): phosphoprotein kinase B (p-PKB/AKT), total-AKT (t-AKT), p-forkhead box O1 (p-FoxO1), t-FoxO1, BCL-2-associated X protein (BAX), lysine 48-linked ubiquitin (K48-Ub), PKG, p-vasodilator-stimulated phosphoprotein (p-VASP), t-VASP, and glyceraldehyde 3-phosphate dehydrogenase (GAPDH). Antiproliferating cell nuclear antigen (PCNA) was obtained from Santa Cruz Biotechnology (Dallas, USA), and anti-Ub was purchased from Proteintech Group (Manchester, UK).

### Animal experiments

Adult male C57BL/6 mice (8–10 weeks old) were purchased from the Institute of Laboratory Animal Science, Chinese Academy of Medical Sciences (Beijing, China) and kept in the Cardiovascular Research Institute of Wuhan University with free access to a standard laboratory chow diet. All mice were maintained in a specific pathogen-free barrier system with a controlled temperature (20–25 °C) and humidity (45–55%) on a regular 12 h light/dark cycle.

Diabetic mouse model was established by intraperitoneal injections of STZ (50 mg/kg) dissolved in a citrate buffer (0.1 mol/L, pH = 4.5) for consecutive 5 days, while the mice assigned to the control groups (Con) were administrated with an equal volume of citrate buffer [38]. One week after the final injection, blood samples were collected from the tail vein and the mice with fasting blood glucose (FBG) > 16.6 mmol/L in three independent measurements were maintained for additional 12 weeks to develop cardiac injury after diabetes induction. To clarify whether OSTN downregulation in diabetic hearts was secondary to AKT inhibition, mice received an intramyocardial injection of adenoviral vectors (Hanbio Biotechnology Co., Shanghai, China) containing the constitutively active AKT (Ad-*ca. Akt*) or a green fluorescent protein (Ad-*Gfp*) at the last 1 week of the study [39, 40]. For adeno-associated virus serotype 9 (AAV9) experiments, mice received a single intravenous injection (1 × 10^11^ viral genomes per mouse) of the cardiotrophic AAV9 (DesignGene Biotechnology, Shanghai, China) carrying the full length of OSTN or negative control (NC) under the cTnT promoter at three weeks after diabetes induction [25, 26]. For proteasome inhibition, mice were intraperitoneally injected with BZM (1 mg/kg, a reversible inhibitor) or CFZ (4 mg/kg, an irreversible inhibitor) once every two days in the final 2 weeks [9, 41]. To verify the involvement of PKG, mice were intraperitoneally administrated with KT5823 (1 mg/kg) once every 2 days for consecutive 6 weeks from the 4th week post-AAV9 infection [26].

To assess the therapeutic potential of OSTN in DCM, diabetic mice were subcutaneously infused with rhOSTN (10 μg/kg/day) or vehicle for 9 weeks. In parallel, diabetic mice were also subcutaneously treated with sildenafil (10 mg/kg/day) alone or in combination with rhOSTN (10 μg/kg/day) for 9 weeks to compare the synergistic effects between OSTN and sildenafil [42]. The functional and molecular parameters were evaluated at 21 weeks after diabetes induction. In another set of experiments, the control mice were infused with rhOSTN (10 μg/kg/day) or vehicle for 9 weeks to determine the hepatotoxic effects of OSTN treatment. All mice were euthanized immediately with an overdose of pentobarbital sodium (200 mg/kg) at the end of each study.

### Echocardiographic and hemodynamic measurements

Cardiac function was measured by the transthoracic echocardiography using a MyLab 30CV ultrasound system (Esaote SpA, Genoa, Italy) and invasive hemodynamic monitoring with a 1.4-French Millar conductance catheter (SPR-839; Millar Instruments, Houston, TX) as described previously [25, 26, 43]. Briefly, mice were quickly anesthetized by 1.5% isoflurane and fixed on the heating pad with a shallow left lateral position. Two-dimensional echocardiography in the parasternal short- or long axis was obtained at the level close to papillary muscles, and then the M-mode images crossing the anterior/posterior walls of the left ventricle were recorded to calculate the functional parameters from at least five consecutive cardiac cycles. Particular attention was given to avoid causing bradycardia or cardiac deformation due to excessive pressure on the chest. After that, a catheter transducer was inserted into the left ventricular cavity through the right carotid artery, and the pressure-volume parameters were monitored by a PowerLab system (AD Instruments Ltd., Oxford, UK).

### Picrosirius red, immunofluorescence, and TUNEL staining

Picrosirius red (PSR) staining was performed to evaluate collagen deposition in murine hearts [44–47]. In brief, heart samples were fixed in 4% neutral formaldehyde for 48 h, dehydrated, and embedded in paraffin, which were then sectioned into 5-μm slices at the middle segments. After deparaffinization and rehydration, cardiac slices were incubated with the PSR solution at room temperature for 90 min, and the average collagen volume was calculated from more than 60 fields per group by the Image-Pro Plus 6.0 analysis system (Media Cybernetics, USA).

Immunofluorescence staining was used to determine OSTN and Ub-positive aggregates in the murine heart [48, 49]. Deparaffinized samples received the high-temperature antigen retrieval process in citric acid buffer and then were incubated with the primary antibodies at 4 °C overnight, followed by the incubation with Alexa Fluor^®^ 488 (Green) or Alexa Fluor^®^ 568 (Red)-conjugated secondary antibodies at 37 °C for 1 h in the dark. Cell nuclei were probed by DAPI and the immunofluorescence images were captured by a DX51 fluorescence microscope (Olympus, Japan).

TUNEL staining was performed using a commercial kit according to the manufacturer’s instructions. Micrographs were captured using the DX51 fluorescence microscope, and cell apoptosis was quantified as the percentage of TUNEL-positive nuclei to total nuclei.

### Western blot and quantitative real-time PCR

Proteins were extracted from the left ventricles or cultured cells using the ice-cold RIPA lysis buffer, and the concentrations were measured by the bicinchoninic acid method. Next, equal amounts of proteins were electrophoresed on sodium dodecyl sulfate-polyacrylamide gels and transferred onto polyvinylidene fluoride membranes. After being blocked in 5% skim milk at room temperature for 1 h, the membranes were probed with the primary antibodies at 4 °C overnight, followed by the incubation with secondary antibodies at room temperature for an additional 1 h [26, 50]. Protein bands were scanned by the ChemiDoc™ XRS + system and analyzed using an Image Lab software (Bio-Rad Laboratories, Inc.). Total RNA was isolated using the TRIzol reagent (Invitrogen, CA, USA) and was reversely transcribed to cDNA with the Maxima First Strand cDNA Synthesis Kit (Roche, Basel, Switzerland). Quantitative real-time PCR was performed on the Roche LightCycler^®^ 480 detection system and gene expressions were normalized to *Gapdh* [48, 51].

### Cell isolation and treatments

Neonatal rat cardiomyocytes were isolated from 1-3-day-old rats according to our previous studies, and bromodeoxyuridine (0.1 mmol/L) was used to inhibit the proliferation of cardiac fibroblasts [44, 50]. After synchronization in serum-free medium for 12 h, the cardiomyocytes were stimulated with high glucose (HG, 25 mmol/L) for 72 h to imitate diabetic injury in vitro, while cells in the matched group were treated with normal glucose (NG, 5.5 mmol/L) containing 19.5 mmol/L mannitol for osmolarity control [38, 52]. To confirm the protective role of OSTN in vitro, cells were treated with rhOSTN (5 μg/mL) or vehicle in the presence or absence of HG stimulation for 72 h [26]. In a separate study, cells were incubated with the small interfering RNA against *Ostn* (si*Ostn*, 50 nmol/L) using a Lipo6000^TM^ transfection reagent for 4 h to knock down endogenous OSTN expression and then cultured in normal medium for 24 h before further intervention [25, 39]. To suppress proteasomal activity, BZM (0.1 μmol/L) or CFZ (1 μmol/L) was added to the culture in the last 24 h [14, 53]. For PKG silence, cells were pre-treated with si*Pkg* (50 nmol/L) for 4 h and then maintained in a normal medium for an additional 24 h before further stimulation. The si*Ostn*, si*Pkg* and corresponding scramble RNA (si*RNA*) were synthesized by RiboBio Co. Ltd (Guangdong, China).

### Determination of proteasomal activity

The proteasomal activity was detected using the commercial fluorometric kits according to the manufacturer’s instructions [41]. Briefly, fresh heart samples or cultured cells were prepared in the ice-cold cell lysis buffer without protease inhibitors or detergents, and then cell debris was removed by centrifugation at 17,000 × *g* for 20 min under 4 °C. The supernatants were immediately used for protein concentration measurements and proteasomal activities determination. The following synthetic fluorogenic peptides: Suc-Leu-Leu-Val-Tyr-AMC, Z-Leu-Leu-Glu-AMC, and Boc-Leu-Arg-Arg-AMC were used, respectively, to measure chemotrypsin-like, caspase-like, and trypsin-like activities at excitation/emission wavelength of 380 nm/460 nm.

### Measurements of the cGMP level and PKG activity

Intracellular cGMP levels were detected based on a competitive ELISA method according to the manufacturer’s instructions. In brief, the supernatants were collected from the ventricular or cellular lysates and then were added to the cGMP monoclonal antibody-coated microwells together with the horseradish peroxidase (HRP)-linked cGMP. After removing the excessive cGMP (test samples or HRP-linked cGMP), tetra-methylbenzidine (TMB) was added to react with HRP at room temperature for 30 min. The absorbance values at 450 nm were recorded for assessing cGMP levels. PKG activity was determined using a recombinant substrate that could be phosphorylated by PKG at specific threonine residuals. Samples were incubated with the recombinant substrate at 30 °C for 30 min. Next, an HRP-conjugated detection antibody against threonine 68/119 substrate was added to incubate with the phosphorylated substrate at room temperature for an additional 1 h, which was then probed by the TMB reagent (30 °C for 10 min) at 450 nm.

### Biochemical analysis

LDH activity was measured by a commercial assay kit according to the manufacturer’s instructions. Fresh heart samples were homogenized in cold assay buffer and centrifuged at 4 °C for 15 min to remove any insoluble material. Tissue supernatants and cell medium were then incubated with the reaction mix, and the absorbance values were measured at 450 nm. Caspase3 activity in the myocardium or cultured cells was measured via detecting the fluorogenic change of Z-DEVD-AMC [26]. Serum levels of alanine transaminase (ALT) and aspartate transaminase (AST) were detected by an ADVIA 2400 automatic biochemical analyzer (Siemens, Tarrytown, USA) [25, 26]. Circulating OSTN concentrations were measured by a sandwich chemiluminescence enzyme immunoassay, as described previously [26, 37]. Briefly, blood samples were quickly stored in microtubes containing EDTA-2Na and then centrifuged at 4 °C for 20 min with the plasma collected. Next, the plasma samples were incubated in pre-coated plates at 4 °C overnight and then probed by an anti-mouse/human OSTN rat antibody. Finally, alkaline phosphatase-conjugated donkey anti-rat antibody was applied to incubate at room temperature for an additional 1 h, and the chemiluminescence signal intensity was measured at 535 nm using a CDP-Star™ substrate with Emerald-II™ enhancer. Cell viability was assessed by the CCK-8 method, as described previously [25, 51].

### Statistical analysis

All experimental groups were randomly assigned without knowledge of the treatments. Treatments, data collection, and processing were performed in blinded manners. All data were presented as the mean ± standard deviation (SD) and analyzed using the SPSS software (Version 22.0). Comparisons between two groups were performed with unpaired Student’s *t* test, whereas differences among three or more groups were evaluated by one-way ANOVA with Tukey post hoc test. A *P* value less than 0.05 was considered statistically significant.

## Results

### OSTN is reduced by AKT/FoxO1 dephosphorylation in diabetic hearts

**Fig. 1:**
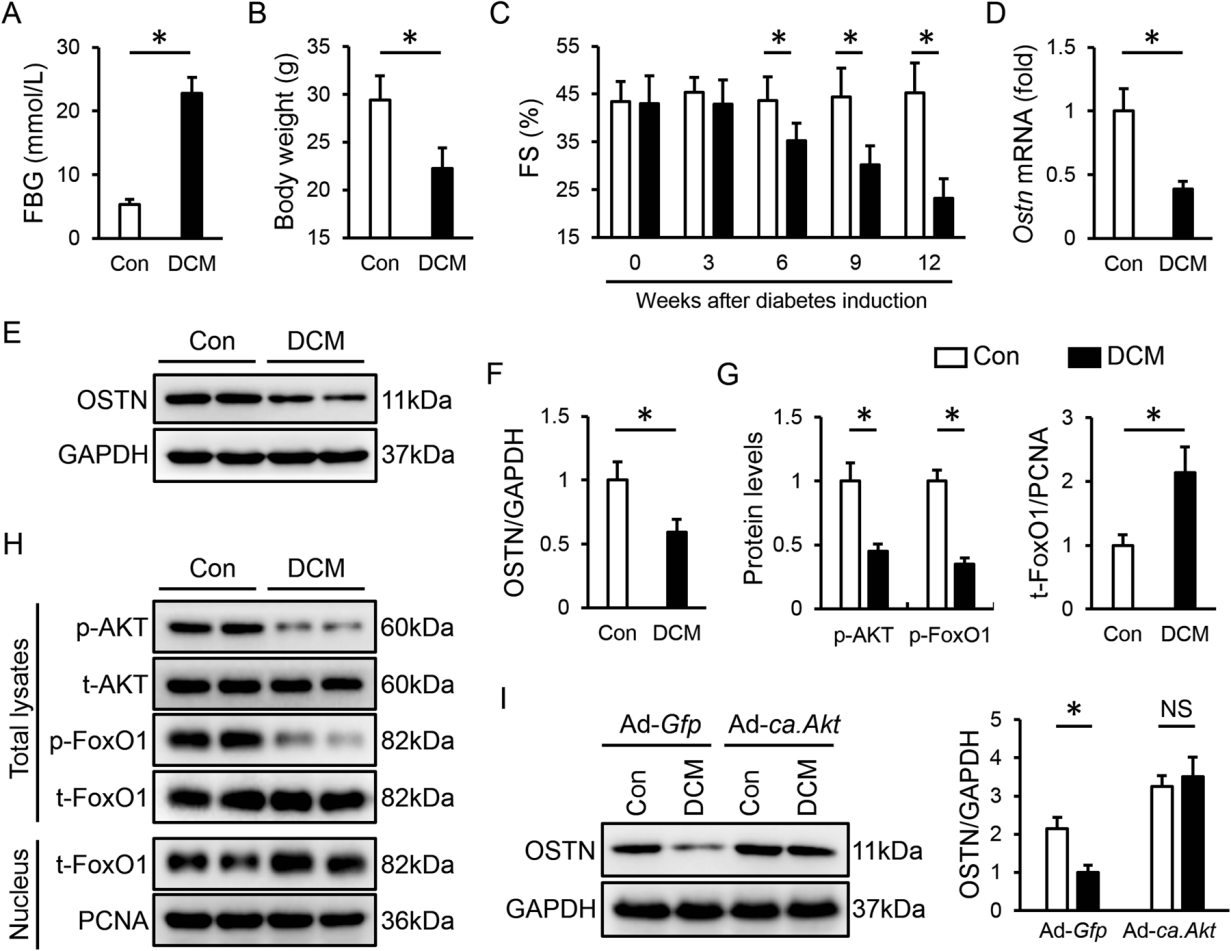
OSTN is reduced by AKT/FoxO1 dephosphorylation in diabetic hearts. **A** FBG levels in mice at 12 weeks after diabetes induction (*n* = 8). **B** Body weight in mice (*n* = 8). **C** FS in mice (*n* = 8). **D** Relative *Ostn* mRNA levels in diabetic hearts (*n* = 6). **E**–**I** Representative western blot images and statistical results (*n* = 6). Data represent mean ± SD. *P < 0.05 versus the matched group, NS indicates no significance.

Mice were exposed to repeated STZ injections and the hyperglycemic mice (FBG > 16.6 mmol/L) were then kept for additional 12 weeks to trigger cardiac injury after diabetes induction. As shown in Fig. 1A, B, diabetic mice exhibited increased FBG levels and decreased body weight. Echocardiographic data identified a contractile impairment in diabetic hearts, suggesting the establishment of DCM (Fig. 1C). To investigate the potential involvement of OSTN in DCM progression, we first detected whether cardiac OSTN expression was altered in diabetic mice. As depicted in Fig. 1D–F, the mRNA and protein levels of OSTN were reduced in diabetic hearts. AKT acts as a critical signaling node in the pathogenesis of DCM that has been linked to the regulation of OSTN via controlling FoxO1 nuclear transport [34]. Therefore, we speculated that the AKT/FoxO1 pathway would be required for OSTN downregulation in diabetic hearts. Consistent with our previous findings, we observed a decreased AKT/FoxO1 phosphorylation in heart samples after STZ treatment, accompanied by a dramatic nuclear accumulation of FoxO1 (Fig. 1G, H) [52]. To verify the role of the AKT pathway, mice received an intramyocardial injection of Ad-*ca. Akt* to overexpress AKT or Ad-*Gfp* as a control at the last 1 week of the study. Western blot results implied that AKT activation blocked OSTN downregulation in hearts upon hyperglycemic stimulation (Fig. 1I and Figure S1A, B). Collectively, we prove that OSTN is reduced by AKT/FoxO1 dephosphorylation in diabetic hearts.

### OSTN attenuates cardiac injury and dysfunction in diabetic mice

**Fig. 2:**
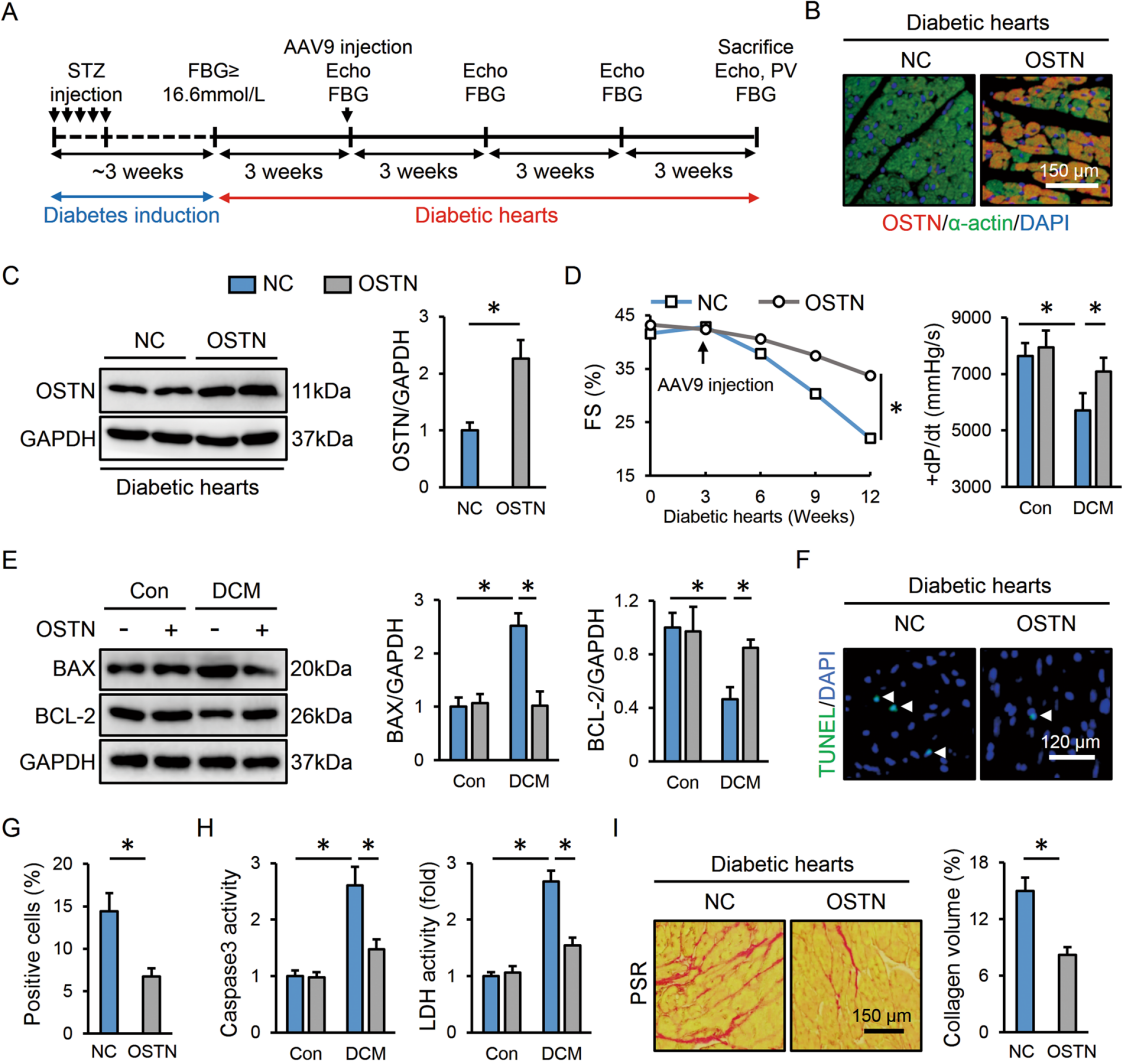
OSTN attenuates cardiac injury and dysfunction in diabetic mice. **A** The treatment schedule. **B** Representative immunofluorescence images of OSTN and α-actin in diabetic hearts with or without OSTN overexpression (*n* = 6). **C** Representative western blot images and statistical results (*n* = 6). **D** Cardiac functional parameters (*n* = 8). **E** Representative western blot images and statistical results (*n* = 6). **F**, **G** Representative TUNEL images in diabetic hearts with or without OSTN overexpression and the quantitative data (*n* = 6). White arrows indicate TUNEL-positive nuclei. **H** Caspase3 and LDH activities in heart samples (*n* = 8). **I** Representative PSR images in diabetic hearts with or without OSTN overexpression and the statistical results (*n* = 6). Data represent mean ± SD. *P < 0.05 versus the matched group.

OSTN reduction in diabetic hearts prompted us to explore whether OSTN overexpression could prevent diabetes-related cardiac injury and dysfunction. Three weeks after diabetes induction, mice received a single tail-vein injection of the cardiotropic AAV9 vectors to specifically overexpress OSTN in the myocardium (Fig. 2A). As shown in Fig. 2B, AAV9 injection for 9 weeks caused a robust and persistent expression of OSTN in murine hearts. Western blot also confirmed the effective transduction and duration of OSTN overexpression, while no alteration of circulating OSTN was observed (Fig. 2C and Figure S2A). Accordingly, cardiac-specific overexpression of OSTN did not affect body weight, food uptake, water consumption, and FBG levels, but resulted in a significant improvement of diabetes-related cardiac dysfunction (Figure S2B–D and Fig. 2D). Chronic hyperglycemic stimulation contributes to cardiomyocyte damage and apoptosis [38, 52]. Consistently, the pro-apoptotic protein, BAX was upregulated, while the anti-apoptotic protein, BCL-2 was downregulated in diabetic hearts, which were prevented by OSTN overexpression (Fig. 2E). TUNEL staining further confirmed the anti-apoptotic effect of OSTN in murine hearts after STZ treatment (Fig. 2F, G). Meanwhile, cardiac caspase3 and LDH activities were both suppressed in diabetic mice with OSTN overexpression (Fig. 2H). Cardiac fibrosis is the other key feature in diabetes-induced cardiac injury [38]. Our PSR staining revealed a distinct collagen deposition in diabetic hearts that was dramatically inhibited by OSTN overexpression (Fig. 2I). PCR analysis further determined the anti-apoptotic and anti-fibrotic effects of OSTN in diabetic hearts, as confirmed by the increased *Bcl-2* and decreased *Bax*, collagen type I alpha 1 (*Col1α1*), collagen type III alpha 1 (*Col3α1*), cellular communication network factor 2 (*Ccn2*, also known as *Ctgf*), and actin alpha 2 smooth muscle (*Acta2*, also known as *α-Sma*) (Figure S2E). Taken together, these findings indicate that OSTN overexpression attenuates diabetes-caused cardiomyocyte apoptosis and fibrotic remodeling, thereby preventing cardiac dysfunction in mice.

### OSTN prevents cardiomyocyte apoptosis and injury upon hyperglycemic stimulation in vitro

**Fig. 3:**
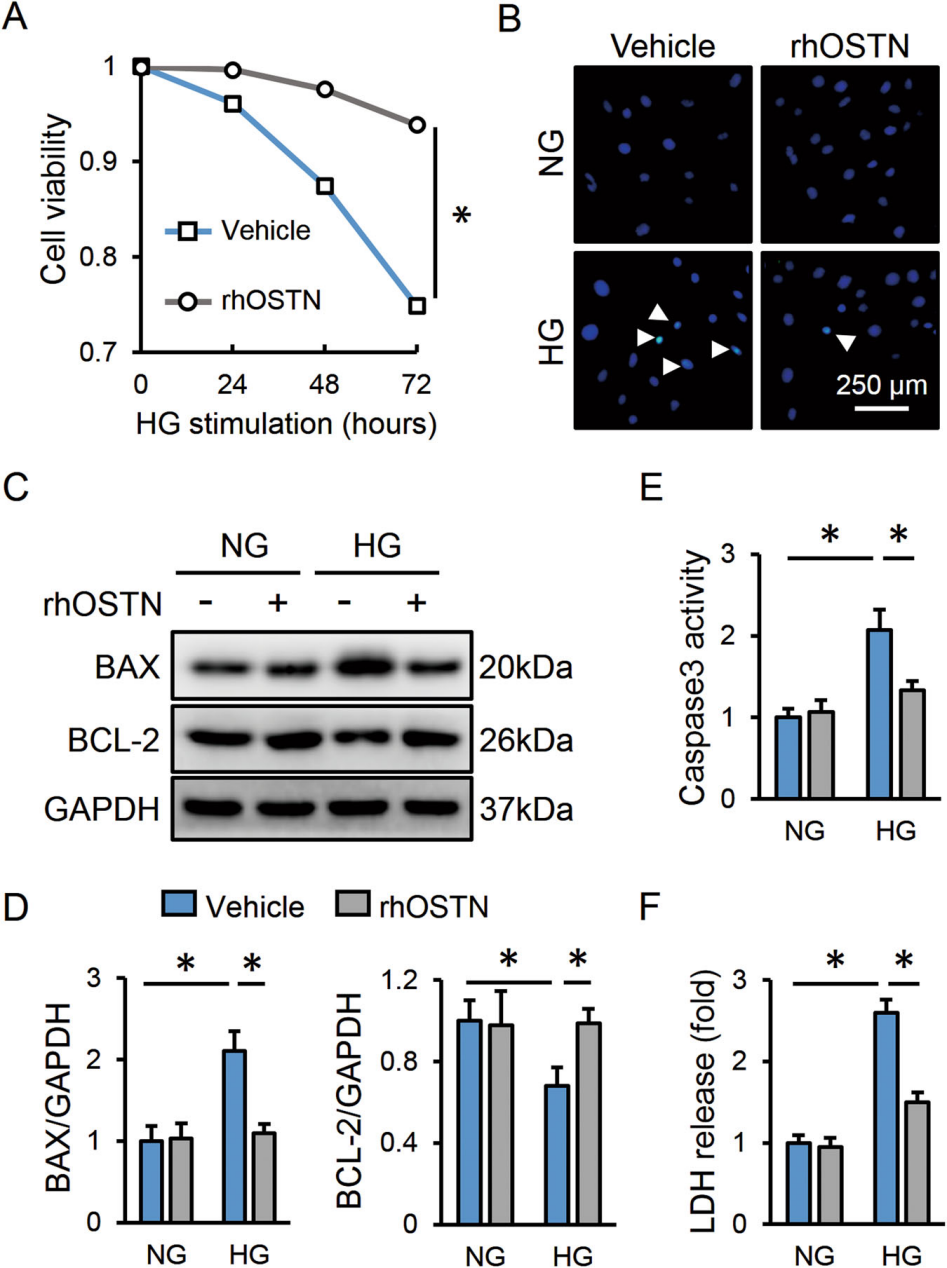
OSTN prevents cardiomyocyte apoptosis and injury upon hyperglycemic stimulation in vitro. **A** Cell viability in neonatal rat cardiomyocytes with or without rhOSTN treatment after HG stimulation (*n* = 5). **B** Representative TUNEL images in cardiomyocytes (*n* = 6). White arrows indicate TUNEL-positive nuclei. **C**, **D** Representative western blot images and statistical results (*n* = 6). **E** Caspase3 activity in cardiomyocytes (*n* = 6). **F** LDH releases to the medium (*n* = 6). Data represent mean ± SD. *P < 0.05 versus the matched group.

We then evaluated the beneficial role of OSTN in vitro by treating neonatal rat cardiomyocytes with rhOSTN or vehicle under HG stimulation. As depicted in Fig. 3A, HG incubation markedly reduced cell survival that was improved by rhOSTN treatment. Accordingly, the cardiomyocytes with HG stimulation had a higher apoptotic rate, but to a less extent in those with rhOSTN protection (Fig. 3B–E). LDH release serves as a biomarker for severe cell injury. As expected, rhOSTN treatment notably decreased LDH releases in HG-stimulated cardiomyocytes (Fig. 3F). Besides, the cardiomyocytes were also transfected with si*Ostn* to knock down endogenous OSTN and then received HG exposure. As shown in Figure S3A, B, OSTN silence exacerbated cardiomyocyte apoptosis upon hyperglycemic stimulation. The cardiomyocytes exposed to HG stimulation had decreased cell viability, and after OSTN silence, cell death was further aggravated (Figure S3C). LDH detection also revealed more severe damage in si*Ostn*-transfected cells than those with si*RNA* treatment after HG insult (Figure S3D). The efficiency of si*Ostn* was verified by PCR data (Figure S3E). However, OSTN expression pattern showed no effects on cardiomyocyte apoptosis and injury under basal conditions. Our data imply that OSTN prevents cardiomyocyte apoptosis and injury upon hyperglycemic stimulation in vitro.

### OSTN improves DCM via restoring proteasomal activity

**Fig. 4:**
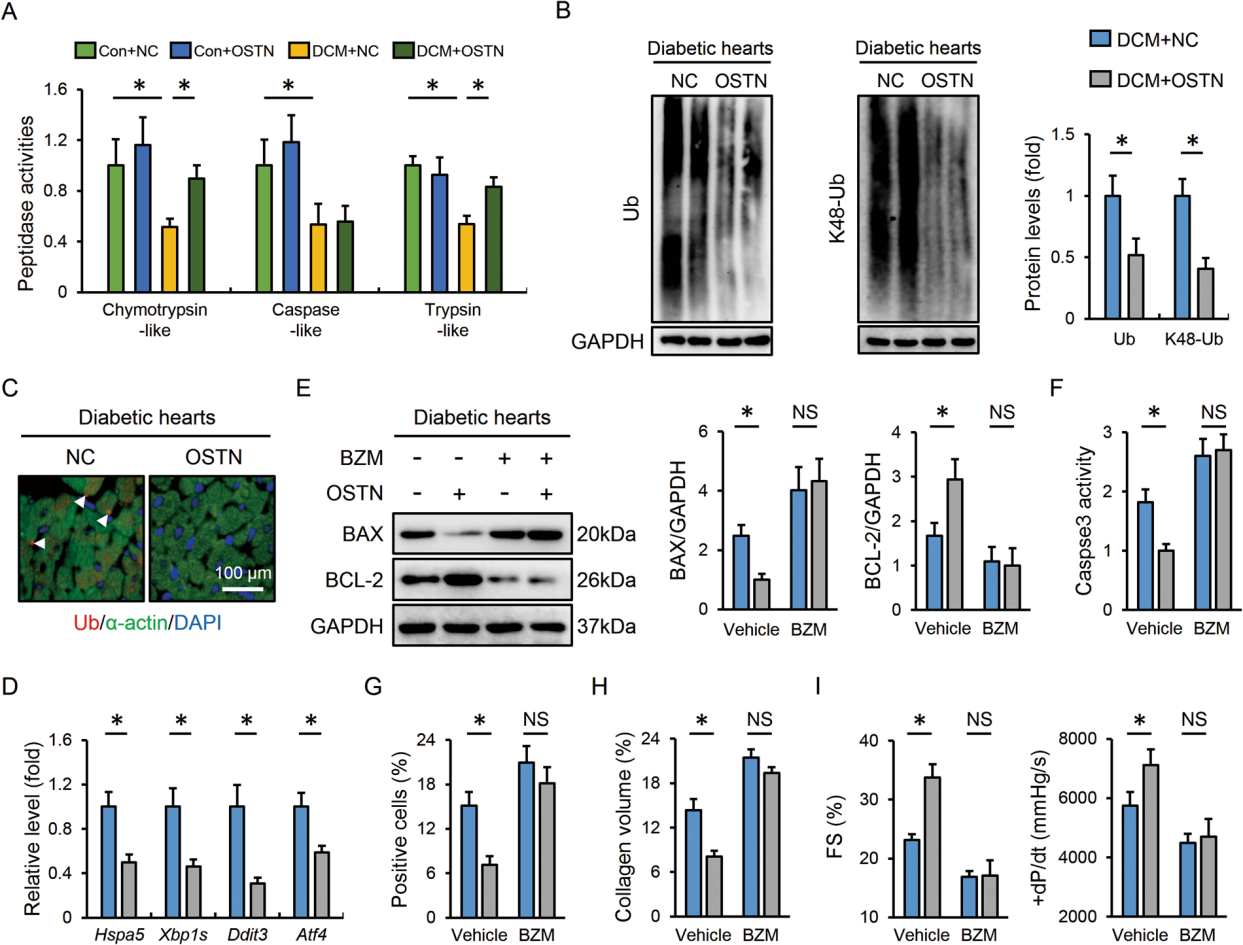
OSTN improves DCM via restoring proteasomal activity. **A** Relative proteasomal activities in heart samples with or without OSTN overexpression (*n* = 6). **B** Representative western blot images and statistical results (*n* = 6). **C** Representative immunofluorescence images of Ub-positive protein aggregates in diabetic hearts with or without OSTN overexpression (*n* = 6). **D** Relative mRNA levels related to endoplasmic reticulum stress in diabetic hearts (*n* = 6). **E** Representative western blot images and statistical results (*n* = 6). **F** Caspase3 activity in heart samples (*n* = 6). **G**, **H** Statistical data of the TUNEL-positive nuclei and collagen deposition (*n* = 6). **I** Cardiac functional parameters (*n* = 8). Data represent mean ± SD. *P < 0.05 versus the matched group, NS indicates no significance.

The proteasome mediates the degradation of most intracellular damaged proteins to ensure proteostasis, while proteasome functional insufficiency causes proteotoxic stresses and cardiac dysfunction [8, 15]. Herein, cardiac proteasomal activity was found to be decreased in diabetic mice that were partially restored after OSTN overexpression, accompanied by the decreases of total and K48-linked ubiquitinated proteins (Fig. 4A, B). Immunostaining results identified fewer Ub-positive protein aggregates in diabetic hearts with OSTN overexpression (Fig. 4C). Impaired protein degradation evoked endoplasmic reticulum stress that was alleviated by OSTN, as evidenced by the decreased mRNA levels of heat shock protein 5 (*Hspa5*, also known as *Grp78* or *Bip*), spliced X-box binding protein 1 (*Xbp1s*), DNA-damage-inducible transcript 3 (*Ddit3*, also known as *Chop*), and activating transcription factor 4 (*Atf4*) (Fig. 4D). To further verify the physiological role of the proteasome in OSTN-mediated cardioprotective effects, mice were treated with BZM to inhibit cardiac proteasome. As expected, BZM administration abrogated the beneficial effects of OSTN on diabetes-related cell apoptosis and fibrotic remodeling (Fig. 4E–H and Figure S4A, B). Of note, the improved cardiac function seen in OSTN-overexpressed mice was completely abolished by BZM treatment, as verified by the unaffected fractional shortening (FS) and the maximum rate of left ventricular pressure rise during isovolumic systole (+dP/dt) (Fig. 4I). Because BZM possesses non-proteasomal actions, we additional used CFZ, a structurally distinct and specific proteasome inhibitor, to suppress cardiac proteasome [54]. As shown in Figure S4C, D, the inhibitory effects of OSTN on cell apoptosis and collagen deposition in diabetic hearts were negated by CFZ injection. Meanwhile, OSTN overexpression failed to prevent diabetes-related cardiac dysfunction in CFZ-treated mice (Figure S4E). These in vivo data identify the necessity of proteasome in the cardioprotection by OSTN.

### Proteasome suppression abolishes the protective effects of OSTN in vitro

In line with the in vivo studies, we observed that rhOSTN increased the proteasomal activity and decreased proteotoxic stresses in HG-treated cardiomyocytes (Figure S5A–C). Conversely, OSTN knockdown further compromised proteasomal function and endoplasmic reticulum homeostasis upon hyperglycemic stimulation (Figure S6A, B). The cardiomyocytes were then treated with BZM to clarify the involvement of the proteasome in vitro. As depicted in Figure S5D, rhOSTN notably inhibited HG-induced cardiomyocyte apoptosis that was abolished in BZM-treated cells. Besides, the increased survival rate and decreased LDH release in the cardiomyocytes with rhOSTN incubation were also abrogated by BZM (Figure S5E). Consistent with the phenotypic alteration, rhOSTN significantly decreased the levels of BAX protein and caspase3 activity and increased BCL-2 protein abundance, yet failed to do so in BZM-treated cells (Figure S5F, G). Further analysis identified that proteasome inhibition by CFZ treatment also blocked the protective effects of rhOSTN against proteotoxic stresses in the cardiomyocytes with HG stimulation, as evidenced by the unaltered cell viability, LDH release, and caspase3 activity (Figure S6C). These results support the conclusion that OSTN protects the cardiomyocytes from hyperglycemic damage via restoring proteasomal activity in vitro.

### OSTN enhances proteasomal activity via activating PKG in vivo

**Fig. 5:**
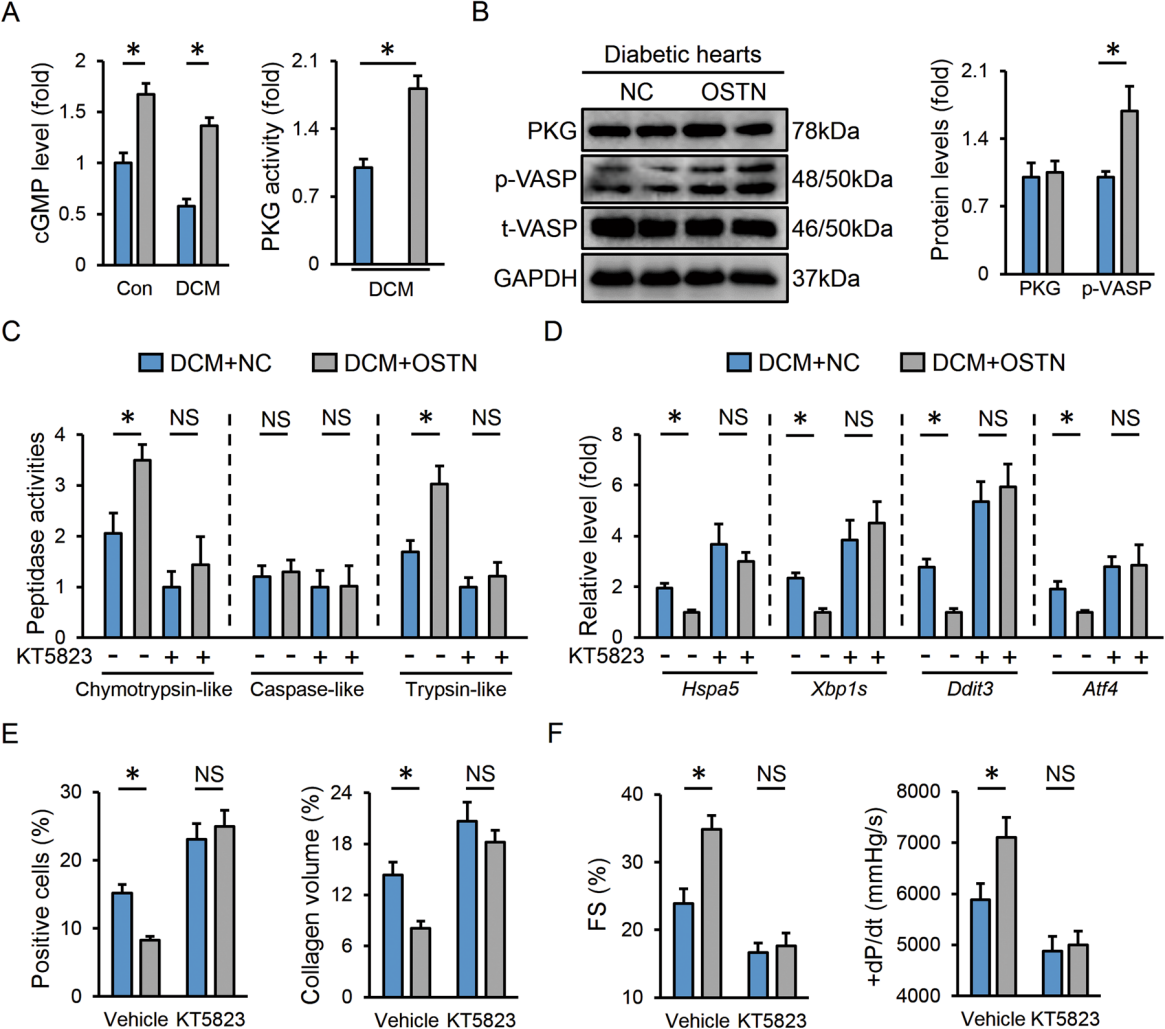
OSTN enhances proteasomal activity via activating PKG in vivo. **A** Relative cGMP levels and PKG activities in heart samples (*n* = 6). **B** Representative western blot images and statistical results (*n* = 6). **C** Relative proteasomal activities in diabetic hearts (*n* = 6). **D** Relative mRNA levels related to endoplasmic reticulum stress in diabetic hearts (*n* = 6). **E** Statistical results of the TUNEL-positive nuclei and collagen deposition (*n* = 6). **F** Cardiac functional parameters (*n* = 8). Data represent mean ± SD. *P < 0.05 versus the matched group, NS indicates no significance.

Proteasomal activity can be modulated at the transcriptional and post-transcriptional levels [7]. We first evaluated the mRNA levels of genes encoding proteasomal 20S subunit (*Psmb1*, *Psmb2,* and *Psmb5*), 19S subunit (*Psmc2 Psmc5* and *Psmc6*), and 11S subunit (*Psme1*), while no change in proteasomal subunit abundance was found in diabetic hearts with or without OSTN overexpression (Figure S6D). Our recent study found that OSTN increased intracellular cGMP levels and PKG activities to prevent doxorubicin-induced cardiotoxicity, we hence investigated whether the restoration of proteasomal activity by OSTN in the context of hyperglycemic stimulation could be ascribed to PKG activation [26]. As shown in Fig. 5A, we observed elevated cGMP levels and PKG activities in diabetic hearts with OSTN overexpression. PKG activation was further confirmed by the higher VASP phosphorylation (a downstream target of PKG); however, no alteration of PKG expression was found (Fig. 5B). To inhibit PKG activity, mice were administrated with KT5823 [26]. The data suggested that KT5823 treatment blunted the restoration of proteasomal activity in diabetic hearts with OSTN overexpression (Fig. 5C). Besides, endoplasmic reticulum functional preservation by OSTN was also absolutely repressed in the presence of KT5823, as indicated by the unaffected mRNA levels of *Hspa5*, *Xbp1s*, *Ddit3*, and *Atf4* (Fig. 5D). Correspondingly, we found that OSTN overexpression protected the diabetic hearts from developing apoptosis and fibrosis, yet failed to do so after PKG inhibition (Fig. 5E). Moreover, OSTN lost its cardioprotective effects in KT5823-treated diabetic mice (Fig. 5F). All the data corroborate that OSTN enhances the cardiac proteasomal activity and subsequently prevents DCM progression via activating PKG.

### PKG silence blocks proteasome activation and beneficial effects by OSTN in vitro

Consistent with the in vivo findings, the cardiomyocytes with rhOSTN incubation had increased cGMP levels and PKG activities (Figure S7A). Meanwhile, an increased VASP phosphorylation instead of PKG expression was found in HG-stimulated cardiomyocytes with rhOSTN incubation (Figure S7B). In contrast, the cells with si*Ostn* transfection exhibited lower cGMP levels and PKG activities upon HG treatment (Figure S7C). Cardiomyocytes were then incubated with si*Pkg* to knock down PKG expression and the efficiency was validated by the decreased PKG protein levels and activities (Figure S7D–F). As shown in Figure S7G, PKG silence abrogated the activation of the proteasome by rhOSTN. Accordingly, the protective effects were also abolished by PKG knockdown, as evidenced by the unaffected cell apoptosis, survival status, and LDH release (Figure S7H, I). These findings define PKG as a molecular node for proteasome activation and cardioprotection by OSTN.

### OSTN is a promising therapeutic target to treat DCM

**Fig. 6:**
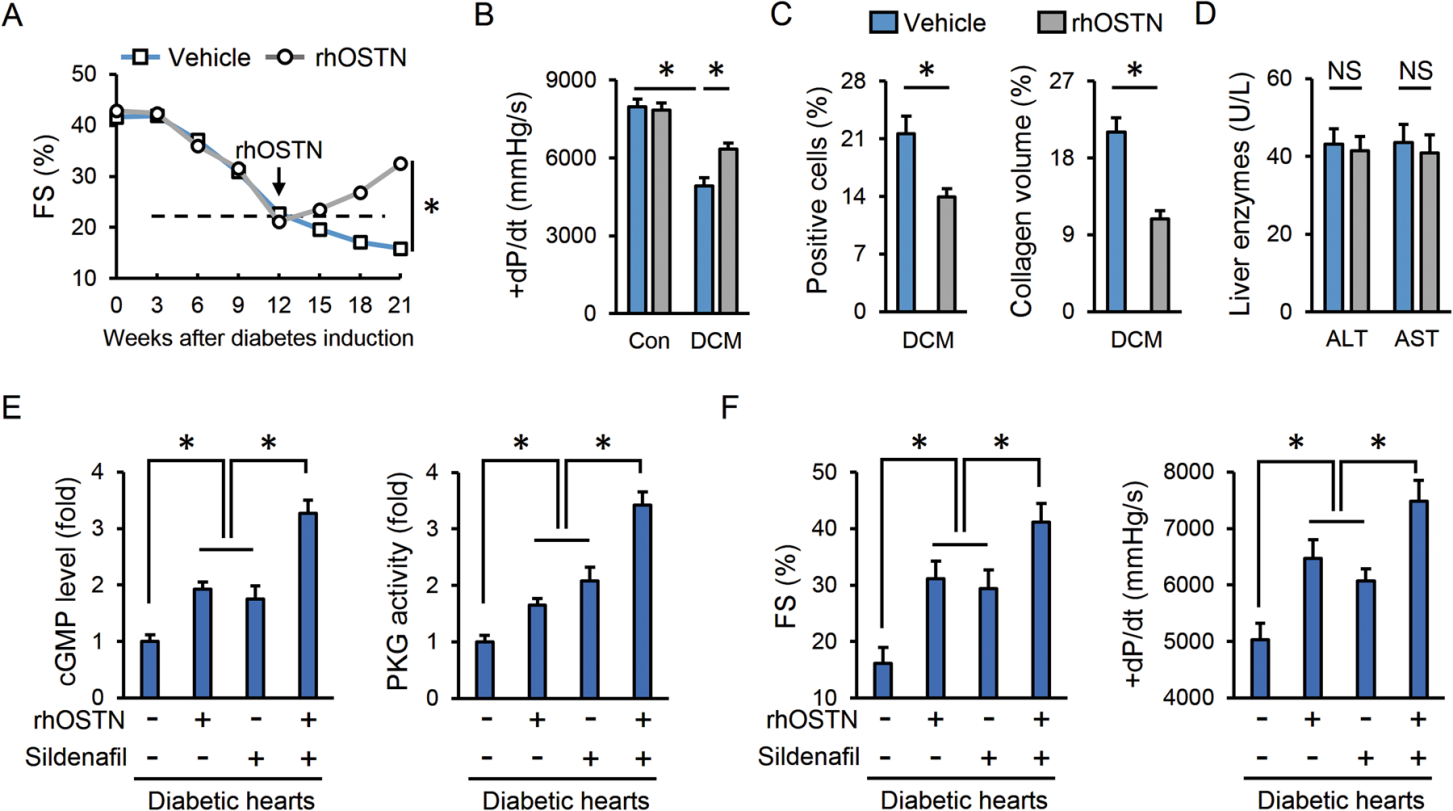
OSTN is a promising therapeutic target to treat DCM. **A**, **B** Cardiac functional parameters (*n* = 8). **C** Statistical results of the TUNEL-positive nuclei and collagen deposition (*n* = 6). **D** Serum levels of liver enzymes (*n* = 6). **E** Relative cGMP levels and PKG activities in heart samples (*n* = 6). **F** Cardiac functional parameters (*n* = 6). Data represent mean ± SD. *P < 0.05 versus the matched group, NS indicates no significance.

Given its cardioprotective capacity, we finally assessed the therapeutic potential of OSTN in pre-established DCM using a recombinant protein. After diabetes induction, mice were maintained for 12 weeks to develop a diabetes-related cardiac injury, which then received rhOSTN or vehicle infusion for additional 9 weeks. Of note, rhOSTN infusion prevented and even partially reversed the progression of cardiac dysfunction in diabetic mice (Fig. 6A, B). Cell apoptosis and collagen deposition were also reduced in diabetic hearts with rhOSTN treatment (Fig. 6C). Moreover, no hepatotoxicity was found in the control mice with systemic exposure of rhOSTN for 9 weeks, as evaluated by the serum levels of ALT and AST (Fig. 6D). We and others found that OSTN increased intracellular cGMP levels and PKG activities to exert the cardioprotective effects [26, 37]. Phosphodiesterase 5 (PDE5) acts as a cGMP-specific phosphodiesterase and blunts the cGMP/PKG pathway via promoting cGMP degradation. PDE5 inhibitors are kinds of agents to prolong the physiological effects of cGMP/PKG signaling that confer multiple benefits to cardiovascular diseases, including DCM in a randomized and controlled trial [55, 56]. We thus compared the cardioprotective capacity between rhOSTN and sildenafil in diabetic mice. As depicted in Fig. 6E, rhOSTN and sildenafil treatment showed comparable effects on cGMP elevation and PKG activation. Besides, co-administration of sildenafil and rhOSTN further increased cardiac cGMP levels and PKG activities in diabetic mice (Fig. 6E). Accordingly, rhOSTN treatment exerted similar roles as sildenafil in treating DCM and had synergistic protection with sildenafil (Fig. 6F). These findings define OSTN as an alternative or at least adjuvant drug to PDE5 inhibitors, especially for those who are unsuitable or unable to tolerate the treatment of PDE5 inhibitors. In conclusion, we propose that OSTN is a promising therapeutic target to treat DCM.

## Discussion

Multiple mechanisms contribute to cardiac impairment in diabetic populations, including oxidative stress, inflammation, and advanced glycation end products [21, 38, 52]. These pathogenic factors subsequently interfere with protein synthesis and degradation, and also disrupt protein qualities via oxidizing, misfolding, or other damaging patterns. Accumulation of the damaged proteins then perturbs endoplasmic reticulum function, which in turn exacerbates proteotoxic stresses and cell injury [12]. The proteasome helps to remove the damaged and/or unnecessary proteins and ultimately minimizes the aggregation and toxicity of these proteins. Several types of proteasome complexes have been identified in eukaryotic organisms and they are generally assembled by a 20S catalytic core particle and different regulatory particles (19S or 11S). Protein degradation occurs inside a narrow proteolytic chamber of the 20S core particle that has caspase-like, trypsin-like and chymotrypsin-like activities residing respectively in β1, β2, and β5 subunits [7]. Proteasome functional insufficiency is observed in several forms of cardiac diseases that cause defective protein quality control and an increased proteotoxic injury. Predmore et al. found that proteasome peptidase activity was impaired in human hypertrophic and failing hearts, but partially restored after mechanical unloading [8]. Proteasome inhibition exacerbated, whereas enhancement of proteasomal activity prevented cardiac ischemia–reperfusion injury [10, 57]. Results from Spur et al. also revealed that inhibiting chymotrypsin-like activity aggravated doxorubicin-induced cardiotoxicity [11]. In addition, proteasome function was proved to be compromised in diabetic hearts, followed by an accumulation of ubiquitinated proteins and protein aggregates [15]. Yet, other studies reported an increased cardiac proteasomal activity upon hyperglycemic stimulation [58, 59]. We speculated that this discrepancy would be attributed to different extent and duration of DCM. Consistently, Li et al. observed a significant increase of cardiac proteasomal activity at the first month after diabetes induction that was reduced at the second month [15]. The temporal kinetics suggest that the cardiac proteasome is activated as a compensatory mechanism at the early stage of diabetes but eventually compromised with diabetes progression. Herein, we observed a decreased proteasome proteolytic activity in diabetic hearts at the end stage, accompanied by the increased levels of ubiquitinated proteins and endoplasmic reticulum stress; while proteasome functional restoration was capable of blocking the proteotoxic injury and cardiac dysfunction in diabetic mice.

OSTN acts as an exercise-responsive myokine that is implicated in bone growth and muscle metabolism [34, 35, 60]. Recent findings from us and other laboratories have validated the cardioprotective role of OSTN in mice with myocardial infarction or doxorubicin insult [26, 37]. Herein, we found that OSTN was downregulated in diabetic hearts secondary to AKT/FoxO1 dephosphorylation. Yasui et al. demonstrated that FoxO1 directly bound to OSTN promoter and inhibited OSTN expression at the transcription level and that the constitutively active form of FoxO1 could markedly reduce OSTN promoter activity and mRNA level in C2C12 myocytes [61]. In addition, Subbotina et al. proved that the AKT/FoxO1 pathway was required for exercise-mediated regulation of OSTN expression in murine skeletal muscle and human primary myoblasts [34]. AKT plays critical roles in DCM progression and insulin treatment is essential for AKT activation. Nishizawa et al. previously determined that insulin directly increased, while the counter-regulators to insulin (e.g., epinephrine, isoproterenol, and forskolin) significantly reduced OSTN expression in myocytes. And OSTN expression was decreased in the skeletal muscle of streptozotocin-treated insulin-deficient mice (lower insulin level), but increased in the muscles of obese insulin-resistant KKAy mice (higher insulin level) [62]. Consistently, we herein detected a decreased cardiac OSTN expression in STZ-induced type 1 diabetes with lower insulin levels. In contrast, OSTN overexpression or infusion attenuated apoptosis, fibrosis, and cardiac dysfunction upon hyperglycemic stimulation. Unlike the acute models by Miyazaki et al. and our recent study, DCM is a long-term chronic disease model. And it is increasingly recognized that the same molecules in different cell types or disease models may differentially regulate global cardiac function [63–65]. Accordingly, we previously found that C1q-tumor necrosis factor-related protein-3 had different effects on cardiac injury upon hypertrophic or hyperglycemic stimulation [48, 52]. Miyazaki et al. primarily examined the cardioprotective effect of circulating OSTN using continuous intravenous infusion of OSTN and the OSTN-transgenic mice [37]. However, systemic OSTN treatment might cause insulin resistance via inhibiting insulin-stimulated glycogen synthesis and glucose uptake in skeletal myocytes [62]. In our previous study and the present one, AAV9-mediated OSTN gene transfer did not affect circulating OSTN level and had no effect on systemic glycometabolism [26]. In combination with the in vitro experiment, we clearly designated a local action of OSTN within the myocardium and cardiomyocytes. OSTN belongs to the natriuretic peptide (NP) family and contains tandem NP-like sequences at the carboxy terminus. This structural identity allows its interaction with the NP clearance receptor (NPR3, also known as NPR-C) to limit endogenous NPs degradation and eventually amplifies the cGMP/PKG pathway [37, 60]. Besides, Chiba et al. found that OSTN could still activate the cGMP/PKG pathway in the presence of a saturated dose of CNP, indicating the existence of NP-independent ways to enhance cGMP/PKG axis [36]. In line with these studies, we proved that OSTN prevented diabetes-related apoptosis, fibrosis, and cardiac dysfunction via restoring proteasome function in a cGMP/PKG-dependent manner. On the contrary, Wang et al reported a cardioprotective effect of MG132 (a proteasome inhibitor) against hyperglycemic stimulation. They found that MG132 could decrease the proteasomal degradation of NF-E2-related factor 2 and inhibitor of nuclear factor κB α, and subsequently preserved cardiac antioxidant and anti-inflammatory capacities [66]. However, proteasome activation by PKG provokes selective degrading pathways for the damaged proteins and does not alter bona fide native endogenous substrates of the proteasome. Besides, MG132 also has some proteasome-independent actions [67]. These findings suggest that PKG-dependent proteasome activation has the potential to treat cardiac diseases with increased proteotoxic stresses. Sildenafil treatment is sufficient to boost cGMP/PKG actions via inhibiting PDE5 that has tremendous benefits to multiple cardiac diseases, including cardiac remodeling, myocardial infarction, DCM, and heart failure [55, 68–70]. Surprisingly, OSTN treatment showed comparable effects with sildenafil on cGMP/PKG activation in diabetic hearts and also exerted synergistic cardioprotection after the co-administration. Of note, STZ injection simply imitates the symptoms of type 1 diabetes in the clinic (e.g., polydipsia, polyphagia, and weight loss); however, whether OSTN can also protect against cardiac dysfunction in a different diabetic model, such as high fat diet-induced type 2 diabetes, remains unclear. Consistent with our present findings, previous studies also detected a significant accumulation of unneeded or damaged proteins in the heart from obese subjects, indicating an impaired proteasomal degradation [71–73]. On the other hand, the cardiac cGMP/PKG pathway is impaired in response to obesity-related type 2 diabetes, and activating the PKG pathway dramatically attenuated pathological cardiomyocyte stiffness and type 2 diabetes mellitus-associated cardiac dysfunction [74, 75]. Certainly, the therapeutic role of OSTN against cardiac dysfunction in a different diabetic model should be further investigated in the future.

Collectively, these findings provide a proof-of-concept that OSTN is a promising therapeutic target for treating DCM.

## Supplementary information

Supplementary material

## Data Availability

All data that support the findings in this study are available from the corresponding author upon reasonable request.
