# Supplementary material for: Osteocrin, a novel myokine, prevents diabetic cardiomyopathy via restoring proteasomal activity
Source: Cell Death Dis. 2021 Jun 16;12(7):624. doi: 10.1038/s41419-021-03922-2 (PMC8209005; doi:10.1038/s41419-021-03922-2)
Supplement: Supplementary file 1 — Supplementary material [file 41419_2021_3922_MOESM1_ESM.docx]

**Supplementary information**

**Osteocrin, a novel myokine,** **prevents diabetic cardiomyopathy via restoring proteasomal activity**

Running title: OSTN prevents diabetic cardiomyopathy

Xin Zhang^1, 2 *^, Can Hu^1, 2 *^, Xiao-Pin Yuan^1, 2 *^, Yu-Pei Yuan^1, 2^, Peng Song^1, 2^, Chun-Yan Kong^1, 2^,

Teng Teng^1, 2^, Min Hu^1, 2^, Si-Chi Xu^1, 2^, Zhen-Guo Ma^1, 2^ & Qi-Zhu Tang^1, 2^

^1^ Department of Cardiology, Renmin Hospital of Wuhan University, Wuhan 430060, China

^2^ Hubei Key Laboratory of Metabolic and Chronic Diseases, Wuhan 430060, China

^*^ These authors contributed equally to this work.

Corresponding author: **Qi-Zhu Tang** and **Zhen-Guo Ma**,

Department of Cardiology,

Renmin Hospital of Wuhan University,

Hubei Key Laboratory of Metabolic and Chronic Diseases,

Wuhan University at Jiefang Road 238, Wuhan 430060, China

Tel.: +86 027-88073385; Fax: +86 027-88042292.

E-mail: [qztang@whu.edu.cn](mailto:qztang@whu.edu.cn) (Qi-Zhu Tang) and [zhengma@whu.edu.cn](mailto:zhengma@whu.edu.cn) (Zhen-Guo Ma).

**
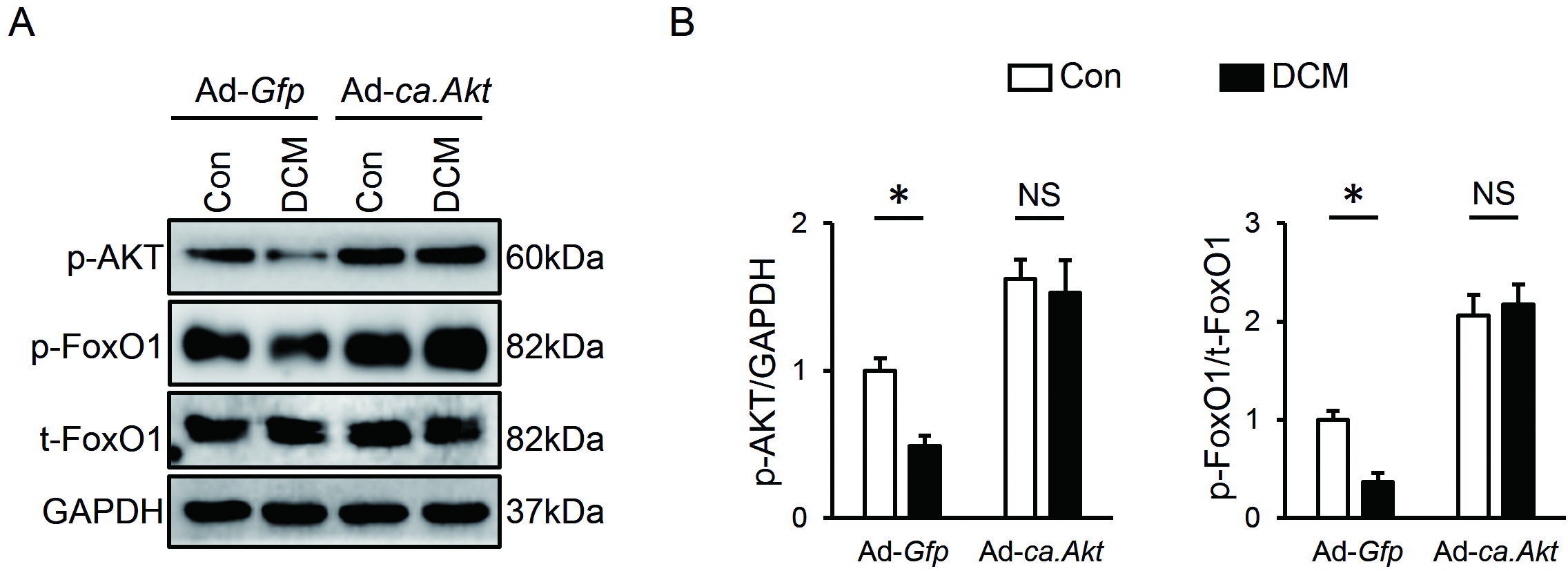
**

**Figure S1. Efficiency of AKT overexpression in murine hearts. (A-B)** Representative western blot images and statistical results (n=6). Data represent mean±SD. **P*<0.05 versus the matched group, NS indicates no significance.


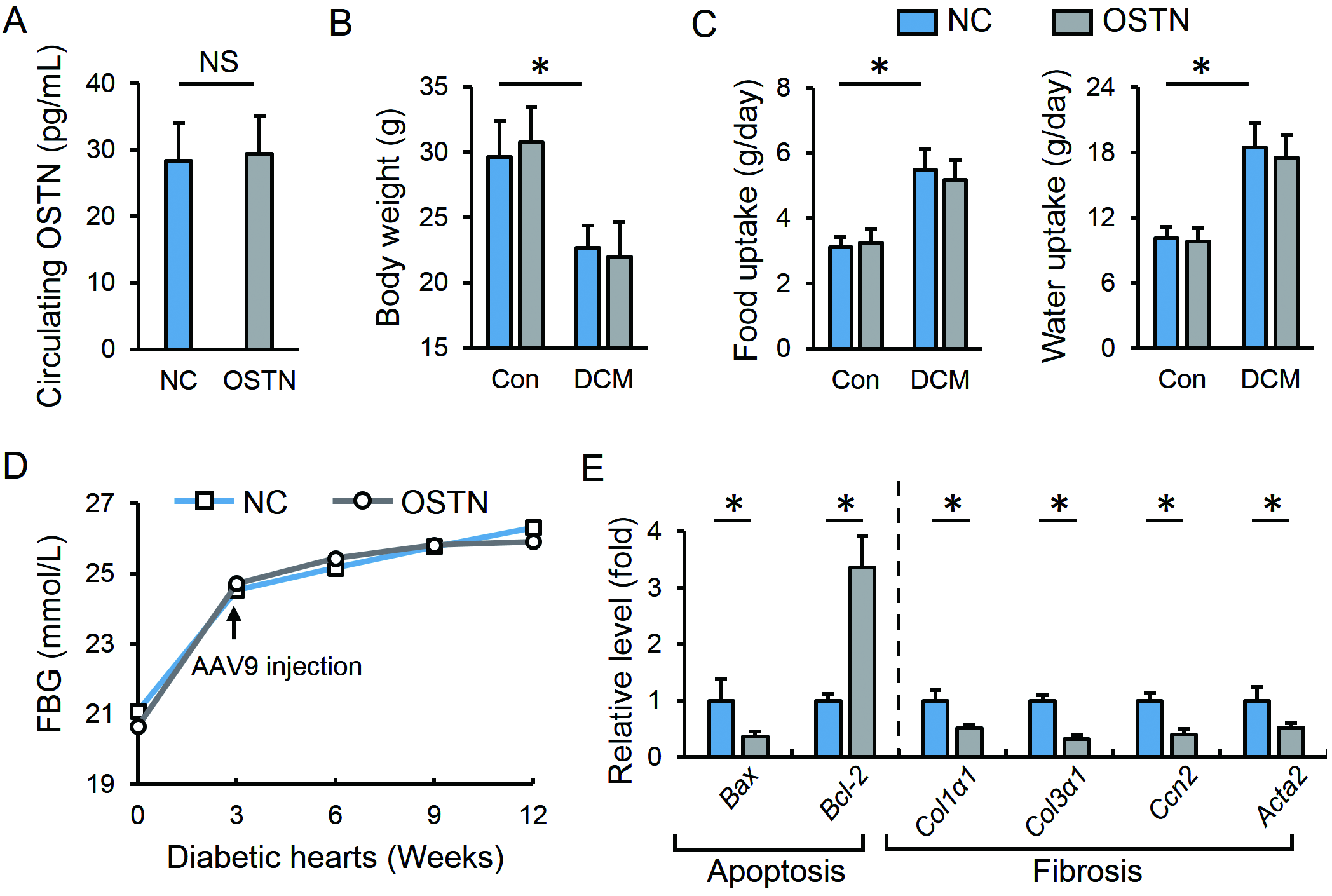


**Figure S2. OSTN attenuates cardiac injury and dysfunction in diabetic mice. (A)** The level of circulating OSTN in mice (n=8). **(B)** Body weight in mice (n=8). **(C)** Food uptake and water consumption in mice (n=10). **(D)** FBG levels in mice after diabetes induction (n=6). **(E)** Relative mRNA levels related to apoptosis and fibrosis in diabetic hearts (n=6). Data represent mean±SD. **P*<0.05 versus the matched group, NS indicates no significance.


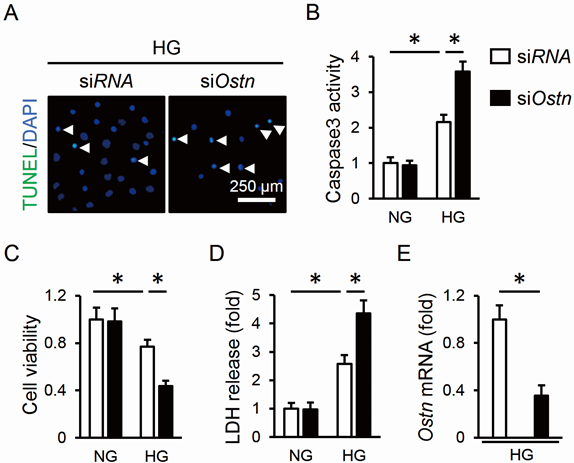


**Figure S3. OSTN silence exacerbates cardiomyocyte apoptosis and injury upon hyperglycemic stimulation in vitro. (A)** Representative TUNEL images in cardiomyocytes (n=6). White arrows indicate TUNEL-positive nuclei. **(B)** Caspase3 activity in cardiomyocytes (n=6). **(C)** Cell viability in neonatal rat cardiomyocytes with or without si*Ostn* transfection after HG stimulation (n=5). **(D)** LDH releases to the medium (n=6). **(E)** Relative *Ostn* mRNA levels in cardiomyocytes with or without si*Ostn* transfection after HG stimulation (n=6). Data represent mean±SD. **P*<0.05 versus the matched group.


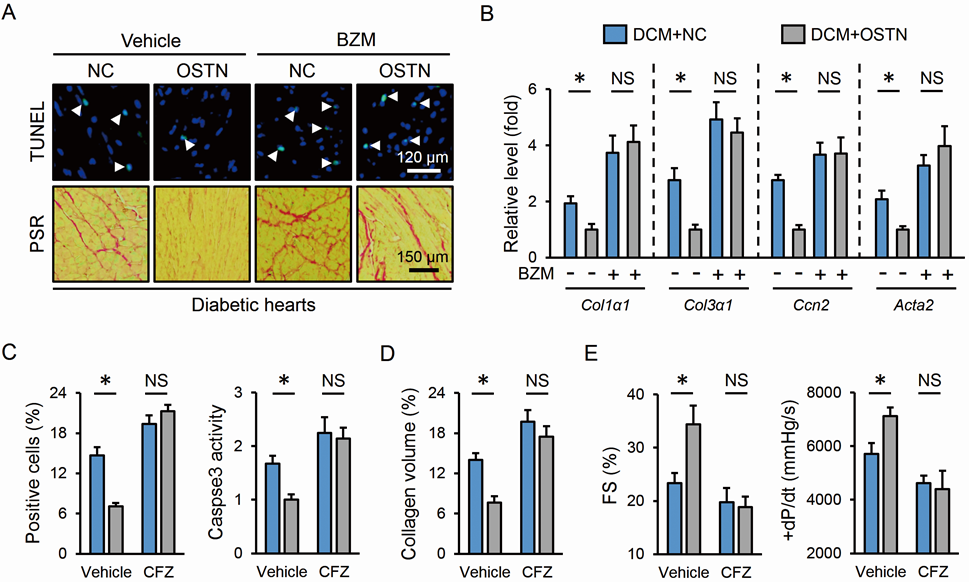


**Figure S4. OSTN improves DCM via restoring proteasomal activity. (A)** Representative TUNEL and PSR images in diabetic hearts (n=6). White arrows indicate TUNEL-positive nuclei. **(B)** Relative mRNA levels related to fibrosis in diabetic hearts (n=6). **(C)** Statistical results of TUNEL-positive nuclei and caspase activity in diabetic hearts (n=6). **(D)** Quantitative data of collagen deposition (n=6). **(E)** Cardiac functional parameters (n=8). Data represent mean±SD. **P*<0.05 versus the matched group, NS indicates no significance.


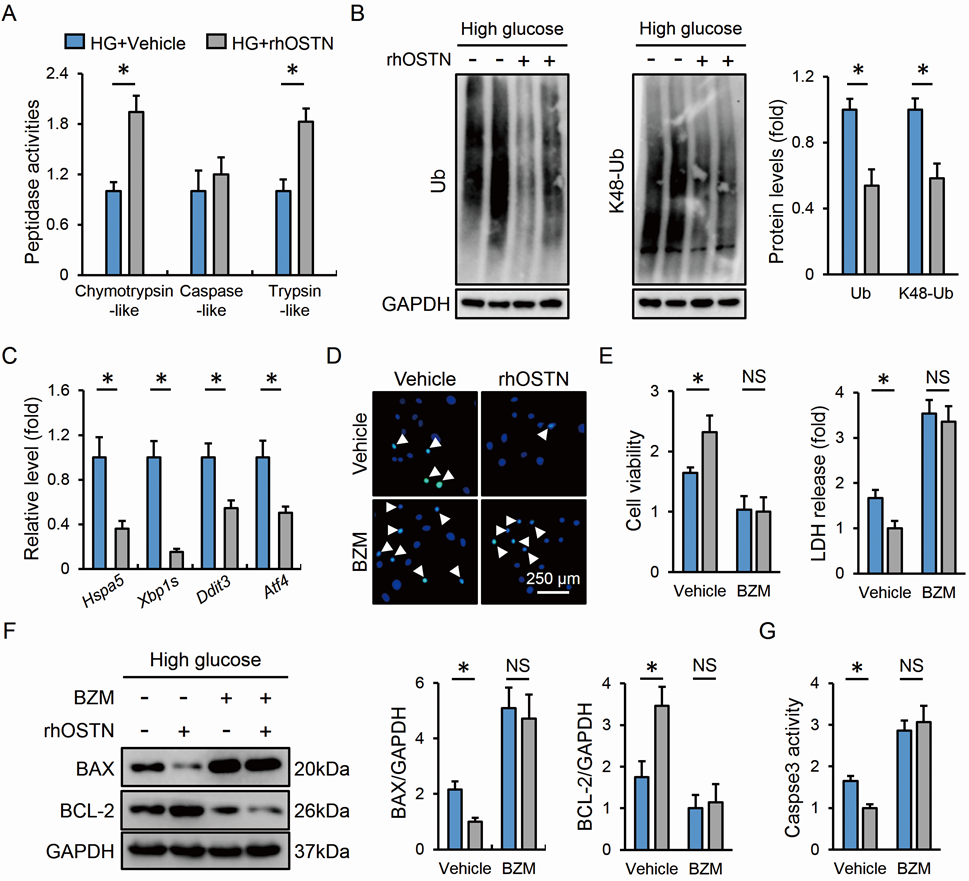


**Figure S5. Proteasome suppression abolishes the protective effects of OSTN in vitro. (A)** Relative proteasomal activities in HG-treated cardiomyocytes with or without rhOSTN protection (n=6). **(B)** Representative western blot images and statistical results (n=6). **(C)** Relative mRNA levels related to endoplasmic reticulum stress in the cardiomyocytes upon hyperglycemic stimulation (n=6). **(D)** Representative TUNEL images in cardiomyocytes (n=6). White arrows indicate TUNEL-positive nuclei. **(E)** Cell viability and LDH releases in HG-treated cardiomyocytes (n=5-6). **(F)** Representative western blot images and statistical results (n=6). **(G)** Caspase3 activity in cardiomyocytes (n=6). Data represent mean±SD. **P*<0.05 versus the matched group, NS indicates no significance.


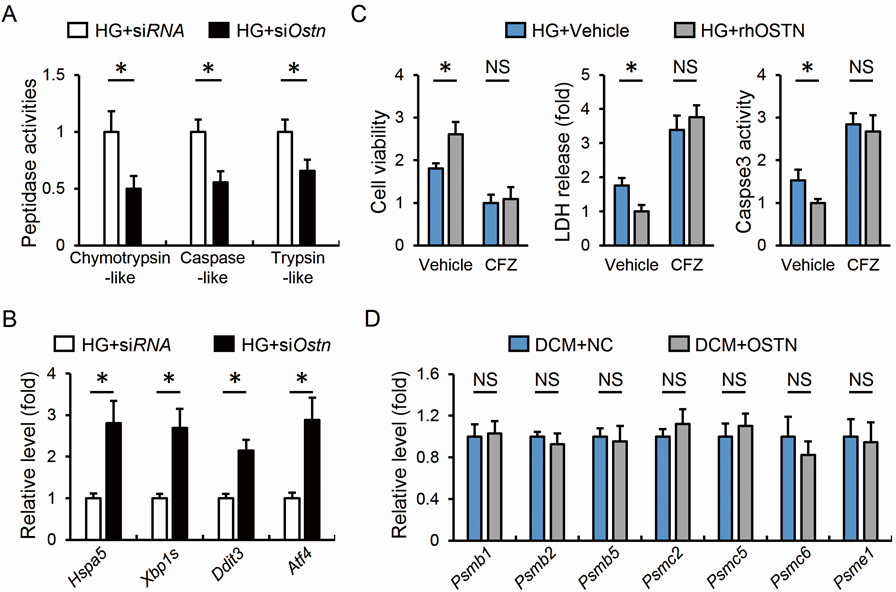


**Figure S6. Role of OSTN on proteasomal activity and abundance. (A)** Relative proteasomal activities in HG-treated cardiomyocytes with or without si*Ostn* transfection (n=6). **(B)** Relative mRNA levels related to endoplasmic reticulum stress in the cardiomyocytes upon hyperglycemic stimulation (n=6). **(C)** Cell viability, LDH releases and caspase3 activity in cardiomyocytes (n=5-6). **(D)** Relative mRNA levels of genes encoding different proteasomal subunits in diabetic hearts (n=6). Data represent mean±SD. **P*<0.05 versus the matched group, NS indicates no significance.


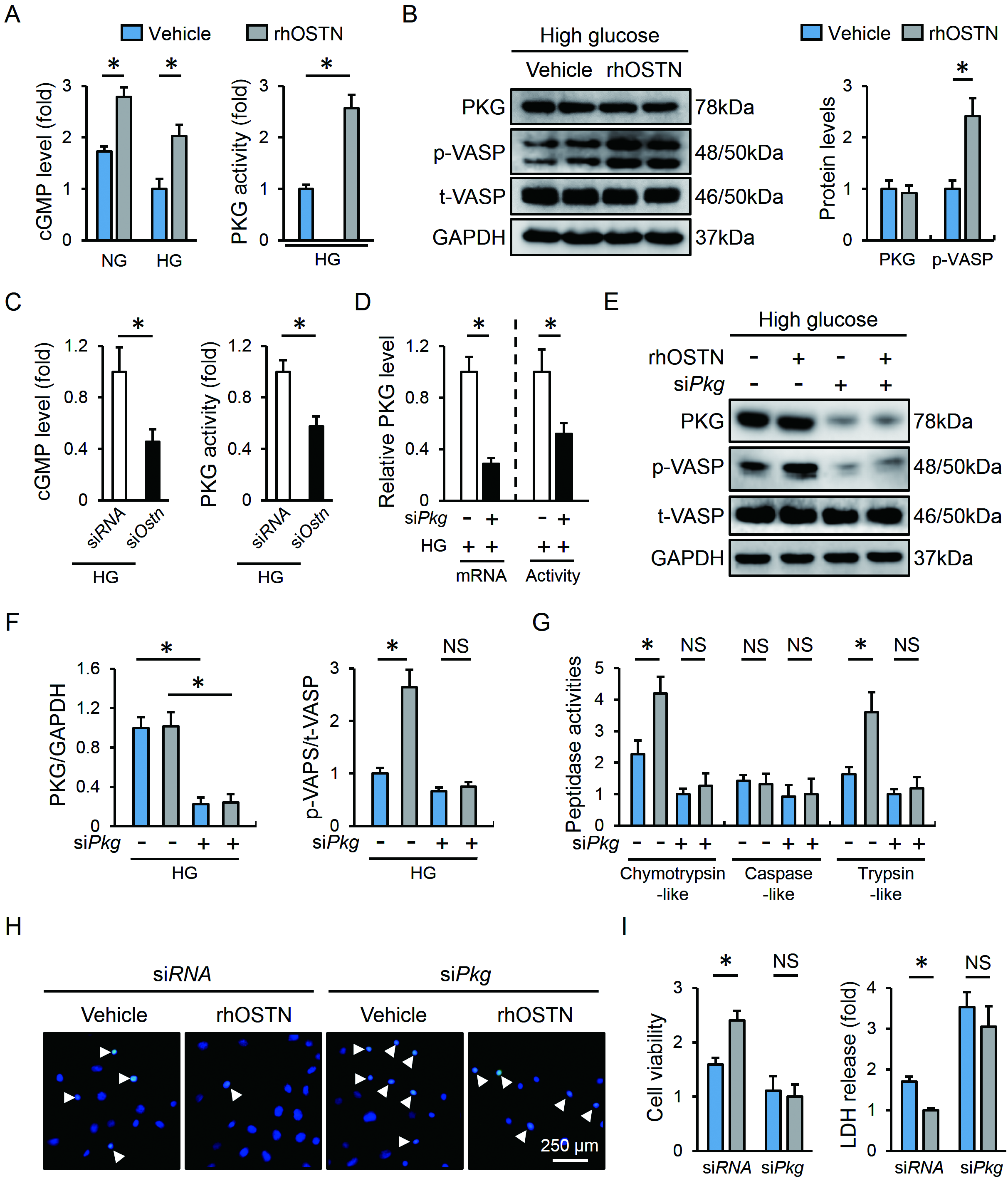


**Figure S7. PKG silence blocks proteasome activation and beneficial effects by OSTN in vitro. (A)** Relative cGMP levels and PKG activities in cardiomyocytes (n=6). **(B)** Representative western blot images and statistical results (n=6). **(C-D)** Relative cGMP levels and PKG activities in cardiomyocytes (n=6). **(E-F)** Representative western blot images and statistical results (n=6). **(G)** Relative proteasomal activities in HG-treated cardiomyocytes (n=6). **(H)** Representative TUNEL images in cardiomyocytes (n=6). White arrows indicate TUNEL-positive nuclei. **(I)** Cell viability and LDH releases in HG-treated cardiomyocytes (n=5-6). Data represent mean±SD. **P*<0.05 versus the matched group, NS indicates no significance.
